# Ultrastructural characteristics of fibrin clots from canine and feline platelet concentrates activated with calcium gluconate or calcium gluconate plus batroxobin

**DOI:** 10.1186/1746-6148-9-77

**Published:** 2013-04-15

**Authors:** Raúl F Silva, Jorge U Carmona, Cleuza MF Rezende

**Affiliations:** 1 Departamento de Clinica e Cirurgia Veterinarias, Universidade Federal de Minas Gerais, Belo Horizonte, Brasil; 2 Grupo de Investigación Terapia Regenerativa, Departamento de Salud Animal, Universidad de Caldas, Caldas, Colombia

**Keywords:** Autologous platelet concentrate, Fibrin clot, Ultrastructural microscopy

## Abstract

**Background:**

The aim of this study was to use transmission electron microscopy to describe the ultrastructural characteristics of clots obtained from canine and feline platelet concentrates (PC) that had been activated with calcium gluconate (CG) or CG plus batroxobin (CGB). Platelets from fibrin clots were classified according their morphological changes. The area of the intercellular space (μm^2^), the area of the fibrin fibers (μm^2^), and the width of the fibrin fibers (μm) were determined for the dog clots. The platelet area (μm^2^), the area of fibrin fibers (μm^2^), the ratio of the minor and major axes of platelets, the ratio of the major and minor axes of platelets, and the number of α-granules found within platelets were measured for the cat clots.

**Results:**

Cat platelets displayed full activation. Dog platelets displayed lysis with loss of normal architecture. In both species, a statistically significant difference was found (P < 0.01) between the fibrin fiber measurements in the PC clots activated with CG and CGB.

**Conclusions:**

The findings suggest that activation with CG caused platelet alpha granules to release their contents. In cats, fibrin production was greater when the PC was activated with CG. In dogs, activation with CG produced thick fibrin fibers.

## Background

After a tissue injury, platelets adhere to the injured blood vessels, which are in direct contact with the exposed collagen in the extracellular matrix. This induces the release of cytokines, growth factors and numerous pro-inflammatory mediators, leading to platelet aggregation and the activation of intrinsic and extrinsic pathways of coagulation and fibrin clot formation [1]. Cellular events that occur during wound healing are initiated, maintained and mediated by a complex series of biochemical events that start with the release of cytokines and growth factors by platelets [2]. There is evidence that the proteins secreted by the platelet α-granules not only produce hemostasis but also induce modulation of inflammation and wound healing [3].

The use of blood-derived products to seal wounds and to stimulate wound healing began with the use of fibrin glues composed of concentrated fibrinogen (polymerization induced by thrombin and calcium) and autologous fibrin glues, which are considered the best choice for avoiding viral infection [4]. Whitman *et al*. [5] described the use of platelet concentrates to improve healing and to replace fibrin glues. Autologous platelet concentrate (PC) preparations constitute a relatively new biotechnology in regenerative medicine. They can be used for the modulation, stimulation and acceleration of tissue healing and bone regeneration in human beings [6,7] and horses [8,9].

Variations in some of the key properties of the PC, including platelet concentration, the type of clot activator, and leukocyte content, can markedly influence the biological effects of this biodrug [10]. The fibrin network supports the platelet and leukocyte concentrate during its application. The density of the fibrin network is mainly determined by the concentration of fibrinogen during PC preparation [11]. Most protocols for PC preparation result in low-density fibrin gels after activation. These kinds of gels are convenient for surgical applications, but they lack a true fibrin support matrix. In contrast, a high-density fibrin network means that the platelet concentrate can be considered a biomaterial [4], and the fibrin matrix itself might have potential healing effects [12].

There is some information available on the electron microscopy characteristics of platelet and fibrin networks in humans [13] and other animal species [14]. However, to our knowledge, there have been few studies on the electron microscopy characteristics of platelet and fibrin networks in clots of PC (in a platelet gel form) from dogs and cats encountered in experimental and clinical settings. This study describes the electron microscopy characteristics of canine and feline PC activated with a non-proteic activating substance (calcium gluconate, CG) or the combination of a proteic activating substance (batroxobin) with calcium gluconate (CGB).

The aim of this study was to use transmission electron microscopy to describe the ultrastructural characteristics of clots (platelet gel) derived from PC collected by the tube method [15,16] and activated with GC or GCB in dogs and cats.

## Results

The average hematological characteristics for dog whole blood were as follows: platelet count 345300/μL, hematocrit 42.2%, white blood cell count (WBC) 16600/μL, lymphocytes 5050/μL, monocytes 790/μL, granulocytes 10700/μL, mean platelet volume (MPV) 9.7 fL and platelet distribution width (PDW) 39.63%. Values for cat whole blood were for platelet count of 412330/μL, hematocrit 33.86%, WBC 10180/μL, lymphocytes 2950/μL, monocytes 630/μL, granulocytes 6620/μL, MPV 7.60 fL and platelet distribution width 34.09%.

The average hematological characteristics for dog PC were as follows: platelet count 529288/μL, hematocrit 4.70%, white blood cell count (WBC) 7391/μL, lymphocytes 3390/μL, monocytes 178/μL, granulocytes 3823/μL, MPV 9.15 fL and PDW 37.60%. The average hematological characteristics for cat PC were as follows: platelet count 863312/μL, hematocrit 1.48%, WBC 4280/μL, lymphocytes 2930/μL, monocytes 120/μL, granulocytes 1230/μL, MPV 9.30 fL and PDW 42.58%.

After activation and incubation, all of the canine platelets displayed lysis and loss of their normal architecture, which corresponds to type III in the classification of platelet morphological changes [17]. Platelet morphological changes in cats included full activation in all platelets, an irregular oval shape with centralization of organelles and the extension of pseudopodia (type II) [17].

**Figure 1:**
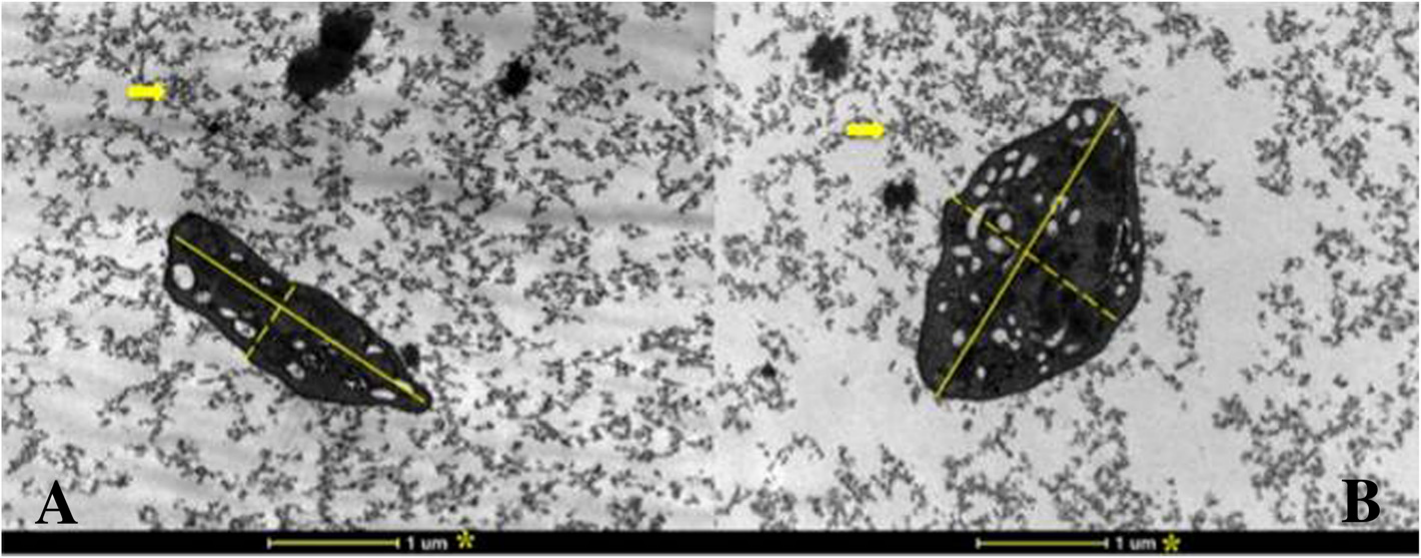
**Transmission electron micrograph showing the major and minor axes in cat platelets activated with calcium gluconate (A) or calcium gluconate plus batroxobin (B).** * Reference, major axes (continuous lines), minor axes (dotted lines). Arrows indicate fibrin. Magnification is 18,000 X.

The samples from the cats showed a statistically significant difference between the areas (μm^2^) or percentage areas of platelets and fibrin fibers, as well as the ratios of the minor and major axis and the ratios of the major and minor axes (Table 1, Figure 1). The median number of α-granules of platelets activated with CG was 4.0 (interquartile range 1.0), and for platelets activated with CGB the median was 8.0 (interquartile range 1.0). These differences were statically significant (P < 0.05) by the Wilcoxon test.

**Table 1:** Ultrastructural characteristics of fibrin clots from feline platelet concentrates activated with calcium gluconate or calcium gluconate plus batroxobin (the data are presented as the mean (standard error))

**Variable**	**Calcium gluconate**	**Calcium gluconate plus batroxobin**
Platelet area (μm^2^)	2.86 (0.24) a	3.79 (0.28)
Platelet area percentage (%)	12.34 (1.05) a	16.36 (1.20)
Ratio of minor-major axes	0.50 (0.03) a	0.60 (0.03)
Ratio of major-minor axes	2.28 (0.13) a	1.86 (0.01)
Area of fibrin fibers (μm^2^)	2.66 (0.17) a	1.58 (0.14)
Percentage area of fibrin fibers (%)	11.50 (0.74) a	6.80 (0.58)

a, denotes statistically significant differences between rows by t-test (P < 0.01).

**Figure 2:**
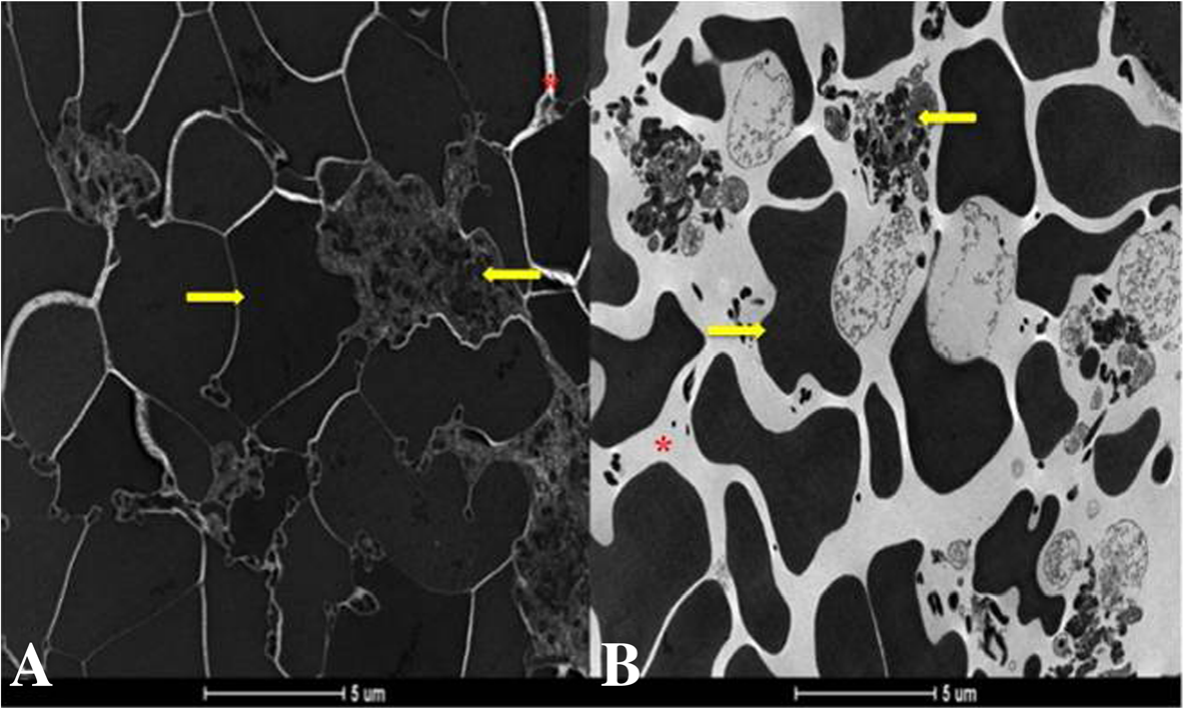
**Transmission electron micrograph showing the characteristics of canine platelet gels activated with calcium gluconate (A) or calcium gluconate plus batroxobin (B). ←** Platelet, **→** Red blood cell, * intercellular space. Magnification is 4,800 X.

**Figure 3:**
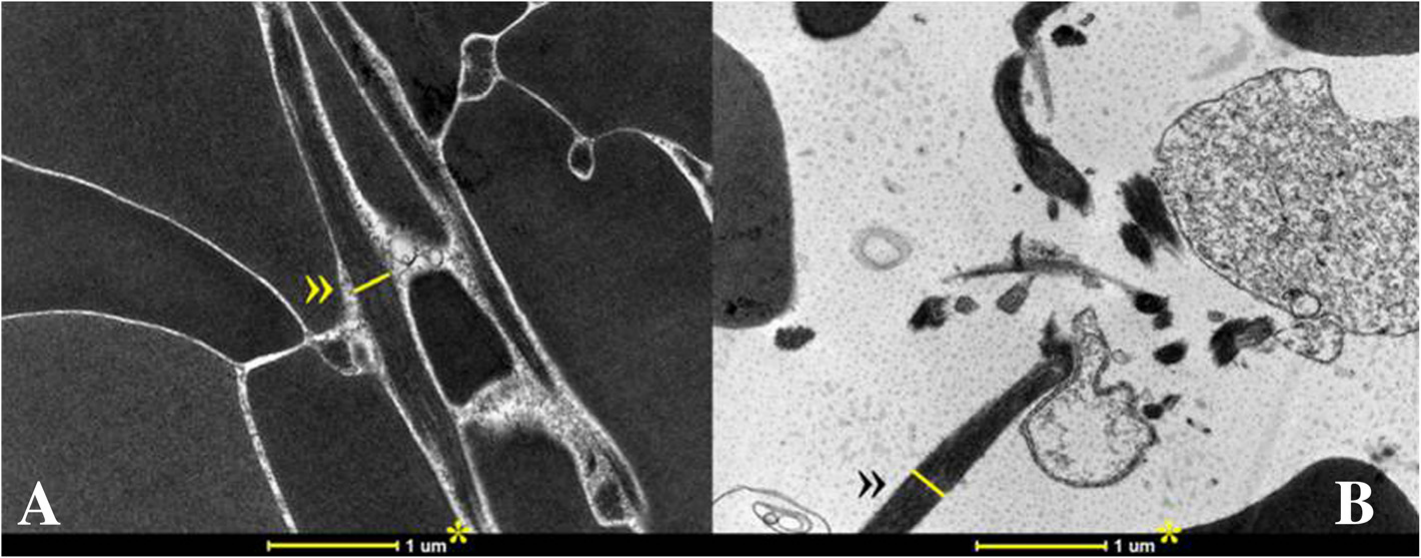
**Transmission electron micrograph showing the width of fibrin fibers in clots of canine PC activated with calcium gluconate (A) or calcium gluconate plus batroxobin (B).** * Reference, » Width of fibrin fiber used for area measurement. Magnification is 18,000 X.

The samples from the dogs showed statistically significant differences between CG and CGB activation (P < 0.01) for the intercellular area (μm^2^), percentage of intercellular space, fibrin fiber area (μm^2^), the percentage area of fibrin fibers and the width of fibrin fibers in clots (Table 2, Figures 2 and 3).

**Table 2:** Ultrastructural characteristics of fibrin clots from canine platelet concentrates activated with calcium gluconate or calcium gluconate plus batroxobin (the data are presented as the mean (standard error))

**Variable**	**Calcium gluconate**	**Calcium gluconate plus batroxobin**
Area of intercellular space (μm^2^)	4.44 (0.22) a	12.83 (0.33)
Percentage area of intercellular space (%)	19.19 (0.95) a	55.41 (1.42)
Area of fibrin fibers (μm^2^)	4.44 (2.19) a	2.64 (1.53)
Percentage area of fibrin fibers (%)	19.18 (9.45) a	11.45 (6.60)
Width of fibrin fibers (μm)	0.2094 (0.0297) a	0.1726 (0.0301)

a, denotes statistically significant differences between rows by t-test (P < 0.01).

## Discussion

The results of this study prevented a comparison between the platelet gels (fibrin clots) obtained from the activated PC of dogs and cats. The ultrastructural differences observed within each species show that there are differences in both platelet response and fibrin polymerization. In addition, the platelets from cats and dogs are quite different. The size of the cat platelets (diameter 2–6 mM, mean platelet volume 8.6-14.1 fL) [18,19] differs from the size of the dog platelets diameter (1–3 mM, mean platelet volume 8.4-11.5 fL) [20,21], and more complex factors such as glycoprotein receptors and signal transduction pathways might be involved in the structural platelet responses observed in our study [22].

According to Sweet *et al.*[23], platelet shape is approximately elliptical, and the ratio between the minor and major axes is 0.5. Mody and King [24] thought that, in general, the elliptical shape is determined by the ratio between the minor and major axes, which is always <1, but when this shape becomes more elongated, the ratio is reversed so that the determination is being made by the ratio between major and minor axes, which is always >1 [24]. In our study, the platelets from cats activated with calcium gluconate show a ratio between minor and major axes of 0.5, and they show a ratio between major and minor axes of 2.28. The cat platelets activated with batroxobin show a ratio between minor and major axes of 0.60 and a ratio between major and minor axes of 1.86, which puts them closer to the elliptical shape. This suggests a greater change of the elliptical shape in platelets activated with calcium gluconate.

Cat platelets activated with CGB occupied a greater percentage of total area than platelets activated with CG, and they also had a greater number of α-granules in their cytoplasm. These findings, along with the relationships between the axes, suggested a moderate degree of platelet activation [23,24].

Platelet gel from dogs activated with CG presented fibrin fiber widths of 209.4 nm and 172.6 nm. These findings are within the range described for thick fibers in horses (150–250 nm), human beings (203–441 nm), monkeys (143–309 nm), oryxes (160–338 nm), sheep (169–286 nm), penguins (182–391 nm) and sea turtles (130–400 nm) [14]. However, Pretorius *et al*. used scanning electron microscopy and did not provide information about the ultrastructural characteristics of the platelets contained in the PRP.

Pretorius *et al*. [14] also described the fine and intermediate fibrin fibers for the aforementioned species. However, these kinds of fibrin fibers were not observed in our study. This may indicate that canine platelet gel from PC activated with either CG or CGB produces only fibrin fibers of higher quality compared with other species (Pretorius *et al*. [14] used thrombin as an activating substance). However, our results show that CGB produces thinner fibrin fibers than those from human PRP activated with thrombin [14]. Our results clearly demonstrate that the activating substance influences the quality of the resulting biomaterial (biodrug).

The smaller area and percentage area of intercellular space observed in the fibrin clots from canine PC activated with calcium gluconate suggest greater cell aggregation. These findings, in conjunction with the greater area and percentage of area of fibrin fibers and the greater width of the fibrin fibers, indicate a strong degree of platelet activation with rapid formation of fibrin clots, and the fibers in these clots are thicker than those from PC activated with CGB. It is important to consider that platelet activation induced by CG is the result of two different signaling events: the direct effect of Ca^2+^ on platelets [25] (after saturation of the calcium antagonist ACD) and the effect of autogenously generated thrombin via Ca^2+^ dependent activation of the coagulation pathway [26].

We are not aware of reports that describe the ultrastructural characteristics of platelet gels from dogs and cats, but the general findings of this study indicate that, regardless of the substance used for PC activation, cat platelets require a longer incubation time than dog platelets to reach the type IV morphological classification [17].

The differences observed between dog and cat clots activated with either CG or CGB could depend on the mechanisms of action of the two activating substances. It is known that batroxobin does not activate platelets trapped within the fibrin network [27]. In this study, however, batroxobin was reconstituted with calcium gluconate, and the amount of calcium was proportionally less when PC was activated only with calcium gluconate. The results observed in this study corroborate the fact that, when batroxobin is used for PC activation, there is no massive release of the contents of platelet alpha granules, in contrast with the effect produced by the calcium salts alone [28].

## Conclusions

The findings of this study suggest that activation with calcium gluconate produces a massive release of alpha granules, at least during the study period (2 hours of incubation after activation). On the other hand, batroxobin prevented early platelet degranulation [27]. Calcium gluconate also produced a better fibrin network than CGB in both species.

The results of this study suggest that PC activated with CG or CGB produces different rates of polymerization, which could cause different speeds of incorporation of the circulating cytokines into the fibrin meshes (intrinsic cytokines). Therefore, the configuration of the fibrin mesh implies differences in the lifespans of these cytokines [29]. This could mean that PC activated with CG produces a thicker fibrin biomaterial and a faster release of GF and cytokines and that PC activated with CGB produces a slow release of growth factors (with a long-term effect).

## Methods

The animal research ethics committee of the Federal University of Minas Gerais approved this study.

### Animals

Four mongrel male animals of each species were used (dogs and cats), and all of the subjects were clinically healthy at the time of blood collection.

### Preparation of autologous platelet concentrates to obtain a fibrin clot

Blood was collected by puncturing the jugular vein in the cats and the saphenous vein in the dogs with a butterfly catheter 21 G (Blood Collection Set, Becton Dickinson and Company Vacutainer, New Jersey, USA). Blood samples were placed in an 8.5-mL tube with 1.5 mL ACD-A (trisodium citrate 22 g/L, citric acid 8 g/L and dextrose 24.5 g/L) (Becton Dickinson and Company Vacutainer, New Jersey, USA). To obtain PC, the feline blood samples were centrifuged (Hettich Rotofix 32A, Tuttlingen, Germany) at 85 g, and the canine blood samples were spun at 191 g for 6 minutes (SIGMA 3 K30, Germany).

### Activation of platelet concentrates

Before activation, PC samples were analyzed using an automated counting device by volumetric impedance (Abacus Junior Vet, Austria). Each sample was analyzed in triplicate. Next, a micropipette with a fixed volume of 1000 μL was used to collect (approximately) the first 100 μL of the red portion and the first 900 μL of plasma below and above the blood-plasma interface, respectively. Samples of each species were divided into two aliquots of 500 μL each, thus forming two groups. The PC samples from one group were activated with 50 μL of calcium gluconate 10% (Ropsohn Therapeutics Ltda, Bogotá, Colombia), and the samples of the other group were activated with 50 μL of batroxobin (Plateltex, Praha, Czech Republic) and reconstituted with 1 mL of calcium gluconate 10%.

### Fibrin clot (platelet gel) preparation for transmission electron microscopy

Two hours after activation, the supernatant was removed from each sample, and the clots were fixed with a “Karnovsky - modified” primary solution (equal parts glutaraldehyde 2.5% and paraformaldehyde 2%) and post-fixed with osmium tetroxide 2% buffered. Subsequently, the fibrin clots were dehydrated in successive passes of 15 minutes in ethanol at concentrations of 50%, 70% (2 times), 85% (2 times), 95% (2 times), and 100% (three times). Finally, they were placed in acetone for 15 minutes (2 times). The fibrin clots were embedded in resin-acetone (1:2) for 1 hour, resin-acetone (1:1) for 1 hour, resin-acetone (2:1) for 12 hours at room temperature, and pure resin for 1 hour, followed by incorporation into molds and storage in a 40°C oven for 1 hour and a 60°C oven for 48 hours. The polymerized samples were used to obtain ultrathin sections of approximately 60 nm, taken once every 300 nm (approximately). The samples were stained for contrast with uranyl acetate and lead citrate. The plates were viewed on a transmission electron microscope (Tecnai G2 Spirit - FEI - 2006, 120 kV). One plate of each sample was evaluated on the transmission electron microscope.

### Evaluation of the micrographs

Microscopic analysis of the 25 micrographs was performed for each plate by taking 100 photomicrographs for each activating substance for each species (200 total for each species). Morphological changes in the platelets of the fibrin clots were initially scored for both species using a scale from 0 to III [17], where 0: unstimulated platelet, slightly oval-shaped, with evenly dispersed organelles in the cytoplasm; I: platelet in uncertain state, rounded profile shape, non-centralized organelles; II: Platelet fully activated, irregular oval with centralization of organelles and extension of pseudopodia; III: damaged platelet, with total lysis and loss of normal architecture.

In addition to the initial classification used for both species, some platelet and fibrin characteristics were independently analyzed for each species. The parameters considered in cats were individual platelet area (μm^2^), percentage of platelet area, fibrin fiber area (μm^2^), percentage of fibrin fiber area, the ratio between the minor and major axes, the ratio between the major and minor axes of platelets, and the number of α-granules found within each platelet (n = 100 platelets for each activating substance).

For the dog samples, the intercellular area (μm^2^), percentage of intercellular space, fibrin fiber area (μm^2^), and percentage of fibrin fiber area were analyzed. This last evaluation was performed in 30 fibrin fibers, including those just over 0.5 mm in length, of which the width was measured in the middle third of each fiber, perpendicular to its central axis. The analyses were performed with a magnification of 18,000 X. The measurements were made with the digital analysis software Image J (Image Processing and Analysis in Java, National Institutes of Health, Maryland, USA).

### Data analysis

For all of the data, a Shapiro-Wilk normality test was performed. The data from the digital analysis software were compared using a Student’s t- test (t) for paired samples, and the number of α-granules was compared using the Wilcoxon test. The threshold for a statistically significant difference was P ≤ 0.01 for all tests.

## Competing interests

Authors declare no competing interests related with this manuscript.

## Authors’ contributions

RFS conceived of the study, collected samples and performed the laboratory tests, performed the statistical analysis and participated in the drafting of the manuscript. CMFR participated in the design and participated in the drafting of the manuscript. JUC coordinated the study, participated in the design and harmonized the drafting of the manuscript. All authors read and approved the final manuscript.
